# MitoSNARE Assembly and Disassembly Factors Regulate Basal Autophagy and Aging in *C. elegans*

**DOI:** 10.3390/ijms24044230

**Published:** 2023-02-20

**Authors:** Ilias Gkikas, Ioanna Daskalaki, Konstantinos Kounakis, Nektarios Tavernarakis, Eirini Lionaki

**Affiliations:** 1 Institute of Molecular Biology and Biotechnology, Foundation for Research and Technology-Hellas, 70013 Heraklion, Crete, Greece; 2 Department of Biology, School of Sciences and Engineering, University of Crete, 71110 Heraklion, Crete, Greece; 3 Department of Basic Sciences, Faculty of Medicine, University of Crete, 71110 Heraklion, Crete, Greece

**Keywords:** mitochondria, SNAREs, autophagy, aging, SYX-17, NSF-1

## Abstract

SNARE proteins reside between opposing membranes and facilitate vesicle fusion, a physiological process ubiquitously required for secretion, endocytosis and autophagy. With age, neurosecretory SNARE activity drops and is pertinent to age-associated neurological disorders. Despite the importance of SNARE complex assembly and disassembly in membrane fusion, their diverse localization hinders the complete understanding of their function. Here, we revealed a subset of SNARE proteins, the syntaxin SYX-17, the synaptobrevins VAMP-7, SNB-6 and the tethering factor USO-1, to be either localized or in close proximity to mitochondria, in vivo. We term them mitoSNAREs and show that animals deficient in mitoSNAREs exhibit increased mitochondria mass and accumulation of autophagosomes. The SNARE disassembly factor NSF-1 seems to be required for the effects of mitoSNARE depletion. Moreover, we find mitoSNAREs to be indispensable for normal aging in both neuronal and non-neuronal tissues. Overall, we uncover a previously unrecognized subset of SNAREs that localize to mitochondria and propose a role of mitoSNARE assembly and disassembly factors in basal autophagy regulation and aging.

## 1. Introduction

In diverse eukaryotic organisms, cellular and intracellular communication is mostly conducted by continuous vesicle formation, transport and fusion events. During formation, vesicles incorporate selected materials (cargos) including lipids, nucleic acids, cytosolic and transmembrane proteins, among others [1]. The culminating actions, membrane-bridging and cargo release require the circulated assembly and disassembly of soluble N-ethylmaleimide-sensitive factor attachment protein receptors (SNARE)-complexes [2,3]. SNAREs can be embedded either in vesicles or in target membranes, and thus will be thereby defined as v- and t-SNAREs, respectively. From a structural perspective, most SNARE proteins have an N domain, consisting of the characteristic heptad repeat region of approximately 60 aa long, known as SNARE motif, and few contain C-terminal transmembrane domains [2]. In mammals, SNARE complex proteins are well-recognized regulators of presynaptic neurotransmitter release as well as endocytic recycling of synaptic vesicles [4,5]. When intertwined at synapse, a parallel four-helix bundle is commonly formed between syntaxin (t-SNAREs), synaptobrevin (v-SNAREs; also known as VAMPs) and SNAP25 (synaptosomal-associated protein, 25 kDa). Synaptotagmins, Rab GTPases and tethering factors such as the long coiled-coil tether P115, also seem to be universally required for SNARE-mediated membrane-bridging [6]. As fusion proceeds, a trans-SNARE complex is formed between adjacent membranes, which is ultimately converted to a cis-SNARE complex where all SNARE proteins become embedded in the acceptor membrane. When membrane fusion is completed, the recycling of SNARE complex components is catalyzed by the ATPase N-ethylmaleimide-sensitive factor 1 (NSF1) and soluble NSF attachment proteins (SNAPs) [7]. NSF1-SNAP-SNARE association interrupts atypical binding events and drives the disassembly of binary and ternary SNARE complexes, thus liberating individual SNAREs and sustaining membrane trafficking [8].

Alternations in individual SNARE protein expression and perturbed complex assembly and disassembly, represent a common feature of cognitive decline pertinent to neurodegenerative diseases, including Alzheimer’s and Parkinson’s [9,10,11]. Even during normal aging, SNARE protein expression in the hippocampal synapses is significantly decreased [12]. It is not known whether this decline is an adaptive mechanism that protects aging neurons or whether it is causal to the aging phenotype. Therefore, despite the central role of synaptic transmission and plasticity in normal aging and age-related diseases, the contribution of SNARE proteins in lifespan regulation remains elusive.

Owing to their diverse localization, it is not surprising that SNAREs’ and SNARE-coupling proteins’ role in membrane fusion has evolved and been adapted for several cellular processes [13]. Arguably, most steps of macroautophagy, a conserved catabolic process that eliminates unwanted subcellular material and organelles through lysosomes, heavily rely on membrane fusion events [14]. Macroautophagy (herein referred to as autophagy) relies on the initiation and expansion of autophagosomal membranes, the fusion of autophagosomes to lysosomes along with vesicular trafficking [15]. Mechanistically, the autophagosomal t-SNARE, syntaxin 17 (STX17) and its cognate v-SNAREs, lysosomal VAMP7 or VAMP-8 as well as synaptosomal-associated protein 29 (SNAP29) come together upon starvation to overcome the energy barrier of autolysosome membrane fusion [16,17]. Recent studies have also shown that autophagosome-lysosome fusion requires additional SNARE protein complexes including that mediated by Ykt6-SNAP29-Syntaxin 7, independently of STX17 [18]. The SNARE disassembly protein NSF1 was shown to have a neuroprotective function in dopaminergic neurons of *Drosophila melanogaster*, by sustaining autophagy upon starvation stress [19]. However, it is unknown whether tethers such as P115 and NSF1 are present on GABA-type-A-associated proteins (GABARAP/LC3 family)-positive autophagic vesicles.

Apart from the mitochondrial localization of STX17 and its diverse functions, few studies have reported the presence of SNAREs on mitochondria. Specifically, the t-SNAREs, syntaxin 4 (STX4) and SNAP23 are localized to mitochondria, and the v-SNARE VAMP1B possesses a mitochondrial targeting signal (MTS) at the C-terminal hydrophobic region, responsible for its mitochondrial localization [20,21,22,23,24]. However, further functional characterization has not been pursued. Considering the identification of multi-localizing and multi-functioning SNARE proteins, several important questions remain unanswered.

The aim of the present study was to identify previously unrecognized mitochondria-localized SNARE proteins and assess their role in autophagy and longevity. In this context, we uncovered a subset of SNARE proteins to be associated with mitochondria. We showed that mitoSNAREs SYX-17, VAMP-7 and SNB-6 regulate mitochondrial abundance through NSF-1 activity. Similarly, we observe the accumulation of autophagosomes upon loss of *syx-17*, *vamp-7* or *snb-6* in an *nsf-1*-dependent manner. Interestingly, neuronal-specific inhibition of mitoSNAREs has detrimental effects on normal aging whilst, in non-neuronal tissues, *uso-1*, and *nsf-1* have a dominant role over *syx-17* and *vamp-7* to regulate lifespan. Our findings suggest an active role of mitoSNARE assembly and disassembly factors in mitochondrial abundance and morphology and propose their engagement in basal autophagy regulation ultimately affecting the aging process.

## 2. Results

### 2.1. A Subset of SNARE Proteins Is Localized to Mitochondria

**Figure 1:**
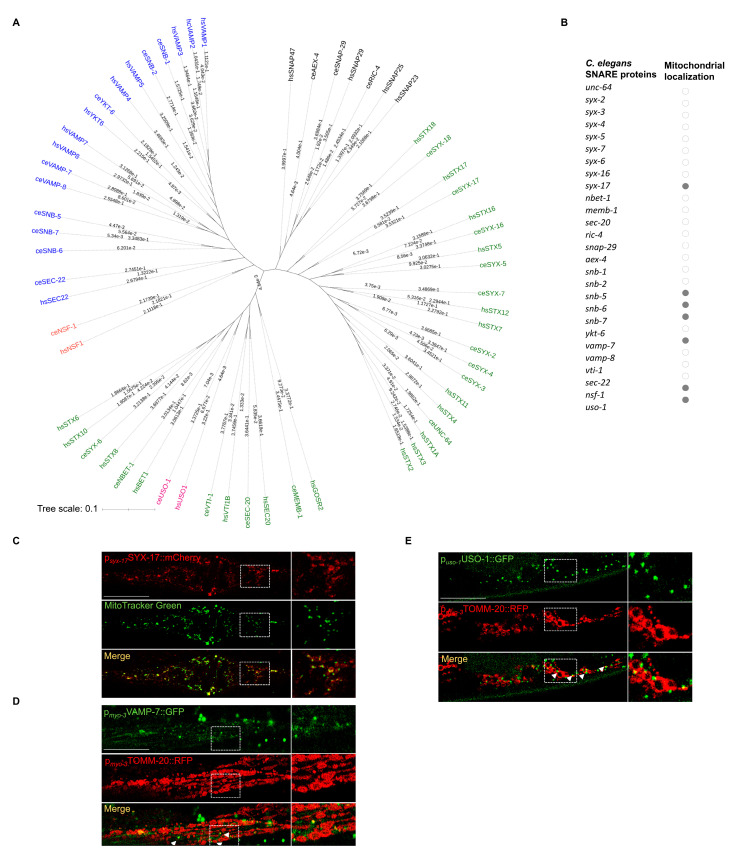
Members of the SNARE protein family are localized to mitochondria. (**A**) Phylogenetic tree of most SNARE and SNARE-coupling proteins between *C. elegans* and humans showing the evolutionary relatedness (v-SNAREs/synaptobrevis/VAMPs are colored blue, t-SNAREs/syntaxins green, tethering factor USO-1 pink, disassembly factor NSF-1 orange). (**B**) List of most SNARE and SNARE-coupling proteins in *C. elegans* that were predicted to be exported to mitochondria (dots marked in grey). (**C**) Transgenic animals expressing *syx-17* fused with mCherry under the control of its own promoter were stained with MitoTracker Green demonstrating that SYX-17 is present in mitochondria (63x lens, scale bar: 20 μm) (MOC = 0.9270 ± 0.006266, M1_(SYX-17)_ = 0.6448 ± 0.09119, M2_(MitoTracker)_ = 0.6350 ± 0.08327). (**D**) Transgenic animals expressing both VAMP-7 fused with GFP and TOMM-20 fused with RFP in muscle cells, demonstrating that a portion of VAMP-7 co-localized with small mitochondrial structures reminiscence of MDVs (63x lens, scale bar: 20 μm) (MOC = 0.8844 ± 0.01490, M1_(TOMM-20)_ = 0.1053 ± 0.02890, M2_(VAMP-7)_ = 0.2876 ± 0.04862. (**E**) Transgenic animals expressing both USO-1 fused with GFP and TOMM-20 fused with RFP in muscle cells showing that USO-1 puncta are in close proximity or on mitochondria (63x lens, scale bar: 25 μm) (MOC = 0.9416 ± 0.01066, M1_(TOMM20)_ = 0.03540 ± 0.005115, M2_(USO-1)_ = 0.1956 ± 0.04986).

Despite the evolutionary variation in SNARE protein sequences, their primary role in vesicular trafficking and membrane fusion is conserved among eukaryotes [25,26,27]. Of interest, the *C. elegans* genome encodes about 30 v-and t-SNARE proteins sharing sequence and functional similarities with their human counterparts [3,28]. To determine the evolutionary relatedness, we constructed the phylogenetic tree of most SNAREs and SNARE-coupling proteins between *C. elegans* and humans (Figure 1A) [29]. The phylogenetic tree displays the orthologous set of SNAREs, which are segregated into four clades including different v-SNAREs, t-SNAREs and SNAPs. Previous complete phylogenetic analysis has also identified a conserved intracellular localization of most SNARE orthologs [30]. As expected, the vast majority are multi-localizing to several subcellular compartments including the ER, Golgi, trans Golgi, endosomes and plasma membrane [25]. Nevertheless, increasing evidence suggests mitochondrial localization of certain SNARE proteins. The first SNARE protein, which was shown to colocalize with human mitochondria, was a specific splice isoform of VAMP-1, namely, VAMP-1B [22]. VAMP-1 is a tail-anchored protein associated with endosomal vesicles with high expression in neuronal tissues. The t-SNARE protein, SYX17, was shown to localize to mitochondria and facilitate the fusion of mitochondria-derived vesicles (MDVs) with endolysosomal compartments in a PINK1/Parkin-dependent and mitophagy-independent manner in mice [31]. Recently, Syntaxin 4 (STX4), known for its plasma membrane localization, was found at/proximal to mitochondria to favor mitochondrial fusion in skeletal muscle cells of mice [24]. Driven by these findings, and with the use of advanced in silico prediction tools for protein localization, we set out to find whether additional SNAREs could be targeted to mitochondria.

In humans, mitochondria consist of approximately 1500 proteins carrying out multiple functions to sustain cell survival [32]. However, the list is far from complete as newly mitochondria-localized proteins are frequently identified [33]. Since 70% of mitochondrial preproteins possess mitochondrial targeting signals (MTS) normally at the N-terminus and infrequently at the C-terminus or internally, many computational methods have been developed for predicting mitochondrial proteins [34]. To discover and annotate novel mitochondrial SNARE proteins, we took advantage of existing and newly developed tools such as DeepL0 2.0, Mitoprot II and iMLP [35,36,37]. For this study, most *C. elegans* SNARE and SNARE-associated proteins were selected and analyzed for the presence or not of conventional MTSs in all possible regions (Figure 1B and Figure S1A–C). Among them, we found a subset of proteins with a high prediction score for mitochondrial localization. Particularly, we found that syntaxin-17 (SYX-17), the *C. elegans* ortholog of the human STX17, similarly to its counterpart, possesses two transmembrane domains at its C-terminus and is predicted by DeepLoc2 to be exported to mitochondria (Figure S1A) [17]. Interestingly, a previously unreported mitochondrial localization for the synaptobrevins SNB-5, SNB-6 and SNB-7, was also predicted by DeepLoc2 (Supplementary Figure S1A). These synaptobrevins display phylogenetic proximity and are the closest *C. elegans* orthologues of the human VAMP3 (Ken Sato, 2013). We also searched for an MTS in VAMP-7/8, the *C. elegans* orthologs of the human VAMP8, and found that only VAMP-7 is predicted by MitoProtII to harbor a presequence cleavage site at 13↓14aa (Figure S1B). So far, numerous proteins with multiple isoforms, each responsible for different subcellular localizations, have been identified [8,38]. In line with this, we found that the SNARE tethering factor USO-1, the *C. elegans* ortholog of the human Golgi protein P115, encodes two protein isoforms USO-1a and USO-1b. Intriguingly, USO-1a has a high probability of mitochondrial targeting and possesses a cleavage site at 63↓64aa as predicted by MitoProtII (Figure S1B). Equivalently, the SNARE disassembly factor NSF-1, the *C. elegans* ortholog of human NSF1, also encodes two protein isoforms NSF-1a and NSF-1b. We revealed that NSF-1a presumably localizes to mitochondria and retains a presequence cleavage site at 23↓24aa as predicted by MitoProtII (Figure S1B). These findings suggest that a subset of SNARE proteins such as the synaptobrevins, USO-1 and NSF-1 can be targeted to mitochondria.

To determine whether the prediction of mitochondrial SNARE (mitoSNAREs) repertory was accurate, we constructed transgenic animals expressing the respective genes fused with GFP, driven by their endogenous or tissue-specific promoters, in case their overexpression could not yield viable transgenic progeny.

Expression of SYX-17::mCherry driven by *syx-17* endogenous promoter is evident in various somatic tissues, including muscles, intestine and neuronal cells (Figure S2A,B). In agreement with previous studies in flies and humans, we find that the *C. elegans* SYX-17 colocalizes with mitochondria both in muscle and neuronal cells (Figure 1C and Figure S2A, respectively) [31,39]. Next, we generated transgenic animals expressing VAMP-7 tagged with GFP in muscle cells and found that a significant portion of VAMP-7 is found in proximity to mitochondria while it co-localizes with small TOMM-20-positive vesicles, reminiscent of mitochondrial-derived vesicles (MDVs)(TOMM-20) (Figure 1D) [31,40]. In addition, SNB-6 tagged with DsRed was also present within body-wall muscle cell mitochondria (Figure S2C). We noticed that animals carrying USO-1 tagged with GFP driven by its own promoter showed strong expression in neuronal and muscle cells (Figure S2D). Previous studies in mammalian cells have reported P115/USO-1 as a tethering factor with an important role in vesicular transport, which operates at the ER-Golgi interface [41,42]. More recently, Golgi-derived vesicles were shown to be recruited to mitochondria constriction sites facilitating mitochondrial fission [43]. Of note, we show that USO-1 vesicle-like structures are localized in close proximity to or on mitochondria, reminiscent of Golgi vesicles found on mitochondria prior to fission events (Figure 1E). Since our sequence analysis revealed that USO-1 harbors a putative mitochondria targeting signal, we cannot exclude the possibility that USO-1 is initially imported and sorted to the outer membrane from where the budding of vesicles can occur (Figure 1B). These findings, combined with the predictions performed above, suggest that a fraction of SYX-17, VAMP-7, SNB-6 and USO-1 localize to mitochondria, under steady-state conditions.

### 2.2. mitoSNARE Proteins Regulate Mitochondrial Morphology and Mass

**Figure 2:**
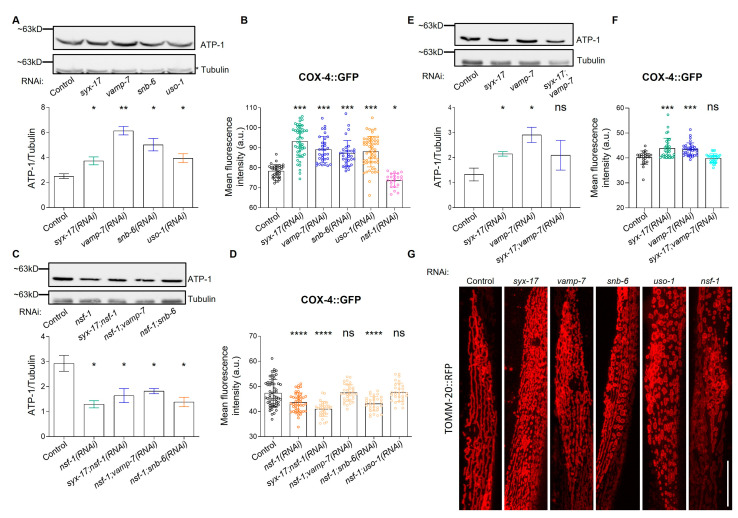
mitoSNAREs regulate mitochondrial abundance in an NSF-1-dependent manner. (**A**) Immunoblot analysis of total ATP-1 protein levels of adult day 1 animals fed with the indicated dsRNA expressing bacteria (upper panel) and the respective quantification of the normalized protein levels (lower panel). *n* = 2 biologically independent experiments (p*syx-17(RNAi)* = 0.0423, p*vamp-7(RNAi)* = 0.0056, p*snb-6(RNAi)* = 0.0215, p*uso-1(RNAi)* = 0.0373, ** *p* < 0.01, unpaired two-tailed *t*-test). (**B**) Quantification of the total COX-4::GFP protein levels in adult day 1 animals fed with the depicted dsRNA expressing bacteria (*n* = 202, p*nsf-1(RNAi)* = 0.03, *** *p* < 0.001, one-way ANOVA). The experiment was performed in 3 biological independent replicates with similar results. (**C**) Immunoblot analysis of total ATP-1 protein levels of adult day 1 animals fed with the indicated dsRNA expressing bacteria (upper panel) and the respective quantification of the normalized protein levels (lower panel). *n* = 2 biologically independent experiments (* *p* < 0.05 unpaired two-tailed *t*-test, ns denotes non-significant differences). (**D**) Quantification of the total COX-4::GFP protein levels in adult day 1 animals fed with the depicted dsRNA expressing bacteria. (*n* = 225, **** *p* < 0.0001, ns denotes non-significant differences, one-way ANOVA). The experiment was performed in 3 biological independent replicates with similar results. (**E**) Immunoblot analysis of total ATP-1 protein levels of adult day 1 animals fed with the indicated dsRNA expressing bacteria (upper panel) and the respective quantification of the normalized protein levels (lower panel). *n* = 2 biologically independent experiments (* *p* < 0.05, ns denotes non-significant differences, unpaired two-tailed *t*-test). (**F**) Quantification of the total COX-4::GFP protein levels in adult day 1 animals fed with the depicted dsRNA expressing bacteria (*n* = 116, *** *p* < 0.001, ns denotes non-significant differences, one-way ANOVA). (**G**) Confocal images showing changes in the mitochondrial network in body wall muscles as observed in animals at day 1 of adulthood (63x lens, scale bar: 20 μm).

So far, our understanding of the role of mitochondrial SNAREs mostly comes from studies on mammalian STX17, where its contribution to essential mitochondrial quality control mechanisms has been appreciated [31,44,45,46,47]. Since we have identified previously unrecognized mitoSNAREs, we, therefore, sought to determine their role in mitochondrial abundance and morphology. In this regard, we found that RNAi-mediated knockdown of *syx-17*, *vamp-7*, *snb-6 or uso-1* (Figure S6) increases mitochondrial mass as evidenced by measuring the protein levels of ATP synthase subunit alpha (ATP-1) and cytochrome c oxidase subunit 4 (COX-4) (Figure 2A,B and Figure S3A) [48]. Similar results were obtained with the transgenic reporter strain, which expresses mitochondrial-targeted GFP in the intestine upon mitoSNARE inhibition (Figure S3B) and from live staining of the RNAi-treated worms with a mitochondrial ROS-specific dye (Figure S3D). On the contrary, inhibition of *nsf-1* significantly reduces mitochondrial mass. Notably, we observed that NSF-1 is indispensable for the effect of SYX-17, VAMP-7, SNB-6 and USO-1 inhibition on mitochondrial mass, since its inhibition cancels the activity of the former, pointing toward a synergistic role between SNARE assembly and disassembly components on mitochondrial mass (Figure 2C,D, and Figure S3C,E,F). To examine whether *syx-17* and *vamp-7* genetically interact as shown in mammals, we concomitantly inhibited *syx-17* and *vamp-7* and found the effects of single depletions on mitochondrial mass to be reversed, suggesting that SYX-17 and VAMP-7 act in parallel pathways to control mitochondrial mass (Figure 2E). Next, we questioned whether mitoSNAREs control mitochondrial morphology, as it has been reported for STX17 in flies and humans [46,47]. To examine changes in mitochondrial morphology, we used transgenic animals expressing TOMM-20::RFP in muscle cells. Importantly, we observed that animals deficient for mitoSNAREs obtained different mitochondrial morphology as compared to their control counterparts (Figure 2G and Figure S3G). Specifically, inhibition of *syx-17*, *vamp-7* resulted in a fragmented or branched mitochondrial network, whilst knockdown of *snb-6*, *uso-1* or *nsf-1* resulted in a circular, swollen and disorganized mitochondrial network. Together, the aforementioned findings suggest that mitoSNARE assembly and disassembly factors affect mitochondrial abundance and morphology.

### 2.3. mitoSNARE Activity Is Required during Basal Autophagy

**Figure 3:**
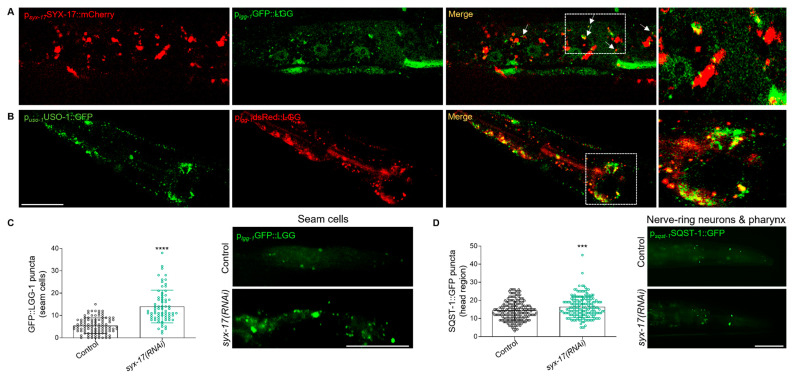
SYX-17 is required for basal autophagy. (**A**) SYX-17 fused with mCherry co-localizes with the autophagosomal protein LGG-1 markedwith GFP (63x lens, scale bar: 80 μm) (MOC = 0.9160 ± 0.01347, M1_(SYX-17)_ = 0.3481 ± 0.07799, M2(LGG-1) = 0,1283 ± 0,04317). (**B**) USO-1 fused with GFP co-localizes with autophagosomal protein LGG-1 (63x lens, scale bar: 50μm) (MOC = 0.9358 ± 0.007102, M1_(LGG-1)_ = 0.4468 ± 0.1296, M2(USO-1) = 0.2256 ± 0.05246). (**C**) Quantification of LGG-1::GFP positive puncta in the seam cells of adult day 1 animals fed with the depicted dsRNA expressing bacteria (left panel) (*n* = 172, **** *p* < 0.0001, unpaired two-tailed *t*-test) and representative epifluorescence images (right panel) (40x lens, scale bar: 25 μm). The experiments were performed in 2 biological independent replicates with similar results. (**D**) Quantification of SQST-1::GFP positive puncta in the head region of adult day 1 animals fed with the depicted dsRNA expressing bacteria (left panel) (*n* = 364, *** *p* = 0.0005, unpaired two-tailed *t*-test) and representative epifluorescence images (right panel) (40x lens, scale bar: 50 μm). The experiments were performed in 2 biological independent replicates with similar results.

Although the presence of SYX-17 on mitochondria facilitating mitochondrial fission was shown to be conserved in nematodes, flies and mammals, its contribution to autophagy has been disputed [39]. Therefore, we sought to determine whether endogenous SYX-17 regulates basal autophagy in *C. elegans.* Initially, we observed that the autophagosomal marker LGG-1/LC3 co-localizes with SYX-17 puncta as evidenced using transgenic animals co-expressing SYX-17 and LGG-1 fused with mCherry and GFP, respectively (Figure 3A). Consistent with the regulatory role of mammalian STX17 on autolysosome fusion, we noted that animals deficient in SYX-17 exhibited an increased number of GFP labeled LGG-1 puncta in hypodermal seam cells, suggesting that autophagosomes are accumulated likely due to inhibition of autolysosome formation (Figure 3C). Since the number of LGG-1 puncta does not predict autophagic activity, we, therefore, monitored the puncta positive for SQST-1::GFP, the *C. elegans* ortholog of human SQSTM1/p62, in the head region. SQST-1/p62 is selectively degraded as it is incorporated into autolysosomes, and its accumulation is indicative of autophagic flux inhibition [49]. We noticed that autophagic activity is blocked upon loss of SYX-17 as it caused an increase in the number of SQST-1-positve structures (Figure 3D). Of note, we found that SYX-17 is not a substrate of autophagy since the loss of LGG-1 reduced the protein levels of SYX-17 (Figure S2E). We also reported that the vesicle tethering factor USO-1 co-localized with LGG-1-positive puncta (Figure 3B). Interestingly, co-expression of SYX-17 with USO-1 under their endogenous promoters (Figure S2F) provides evidence for SYX-17 function at autophagosomes, likely to help mediate autophagosome-lysosome fusion. Taken together, our results support the notion that SYX-17 regulates autophagy in *C. elegans* and propose that the vesicle tethering factor, USO-1 decorates autophagosomes and may have an active role in autophagy regulation.

**Figure 4:**
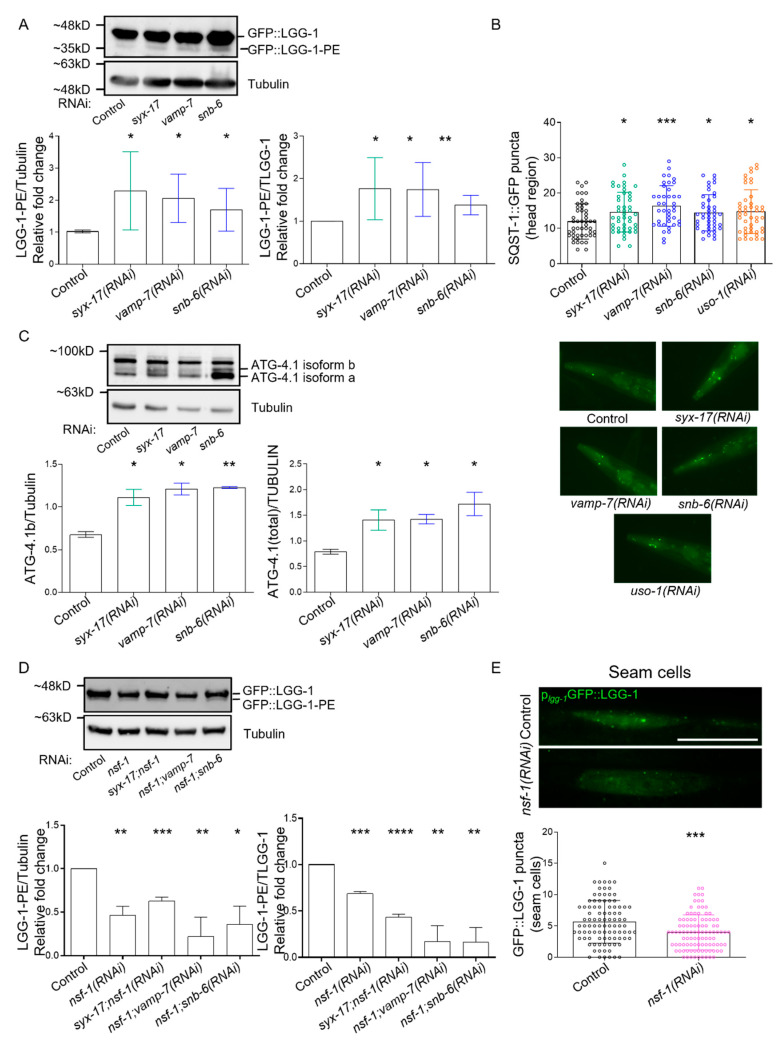
mitoSNARE depletion blocks basal autophagic flux in an NSF-1-dependent manner. (**A**) Immunoblot analysis of total GFP::LGG-1 and GFP::LGG-1-PE protein levels of adult day 1 animals fed with the indicated dsRNA expressing bacteria (upper panel) and the respective quantification of the normalized protein levels (lower panels). *n* = 2 biologically independent experiments (* *p* < 0.05, ** *p* < 0.01, unpaired two-tailed *t*-test). (**B**) Quantification of SQST-1::GFP positive puncta in the head region of adult day 1 animals fed with the depicted dsRNA expressing bacteria. *n* = 2 biologically independent experiments (*n* = 224, * *p* < 0.05, *** *p* < 0.001, unpaired two-tailed *t*-test). Representative images are shown below the graph. The experiments were performed in 2 biological independent replicates with similar results. (**C**) Immunoblot analysis of total ATG-4.1GFP protein levels of adult day 1 animals fed with the indicated dsRNA expressing bacteria (upper panel) and the respective quantification of the normalized protein levels (lower panels). *n* = 2 biologically independent experiments (* *p* < 0.05, ** *p* < 0.01, unpaired two-tailed *t*-test). (**D**) Immunoblot analysis of total GFP::LGG-1 and GFP::LGG-1-PE protein levels of adult day 1 animals fed with the indicated dsRNA expressing bacteria (upper panel) and the respective quantification of the normalized protein levels (lower panels). *n* = 2 biologically independent experiments (* *p* < 0.05, ** *p* < 0.01, *** *p* < 0.001, **** *p* < 0.0001 unpaired two-tailed *t*-test) (**E**) Quantification of LGG-1::GFP positive puncta in the seam cells of adult day 1 animals fed with the depicted dsRNA expressing bacteria (*** *p* < 0.001, unpaired two-tailed *t*-test) (40x lens, scale bar: 20 μm).

To further investigate the role of additional mitoSNARE assembly proteins in autophagy, we employed changes in lipidated and unlipidated LGG-1 levels, as an indication of autophagy status. In line with SYX-17, loss of VAMP-7 or SNB-6 triggered elevation of the LGG-1 lipidated/unlipidated ratio, while the number of SQST-1 punctate structures were increased, suggesting inhibition of autophagic activity (Figure 4A,B and Figure S4A). Moreover, SYX-17 depletion attenuated the cleavage of the dual fluorescent protein fused to LGG-1 in lysosomes, supporting the idea that autophagic flux is reduced under these conditions (Figure S4B) [50,51]. These findings are consistent with the increase in mitochondrial abundance, which is evident upon SYX-17, VAMP-7 or SNB-6 depletion (Figure 2A,B). Furthermore, in animals lacking SYX-17, VAMP-7 or SNB-6, we observed increased protein levels of ATG-4.1, the *C. elegans* ortholog of human ATG4A and ATG4B, which is essential for autophagy initiation and LGG-1 delipidation upon starvation (Figure 4C) [52]. This finding supports the notion that mitoSNAREs play a generalized role in autophagy in addition to autolysosome fusion regulation. We then asked whether the mitoSNARE disassembly factor NSF-1 also regulates autophagy. Importantly, we uncovered that in animals lacking NSF-1, the ratio of lipidated/unlipidated LGG-1 was reduced. The same animals displayed decreased number of LGG-1-decorated autophagosomes in seam cells, together suggesting that basal autophagic activity is suppressed in these animals (Figure 4D,E). Surprisingly, the increase in autophagosome accumulation observed above upon depletion of mitoSNARE assembly proteins was completely abolished by concomitant depletion of both mitoSNARE assembly and disassembly factors (Figure 4C and Figure S4C). Overall, these findings suggest that inhibition of either *syx-17*, *vamp-7* or *snb-6* leads to the accumulation of autophagosomes likely due to blockage of autophagic flux, while depletion of NSF-1 significantly suppresses autophagosome formation and basal autophagic activity.

### 2.4. mitoSNAREs in Control of Aging in C. elegans

**Figure 5:**
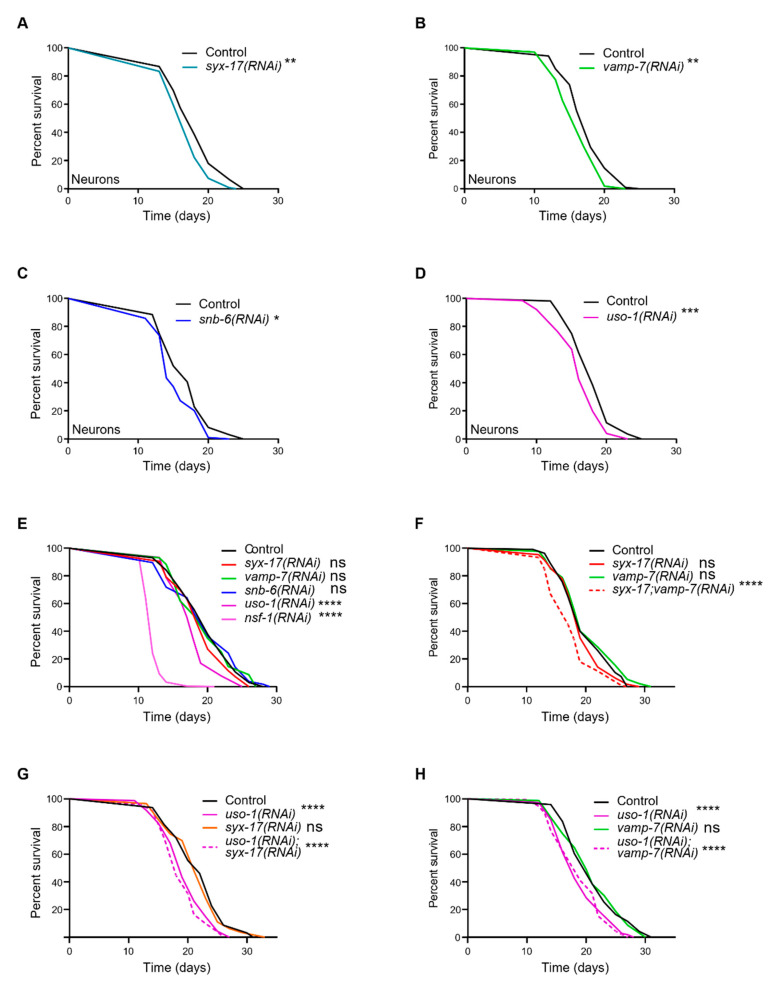
mitoSNARE are essential for normal aging in *C. elegans.* (**A**–**D**)Neuronal-specific inhibition of mitoSNAREs reduces lifespan of otherwise wild-type animals. (**E**) In non-neuronal tissues, knockdown of syx-17, vamp-7 and snb-6 does not cause any changes in lifespan while *nsf-1* and *uso-1* RNAi-silencing causes significant lifespan shortening. (**F**–**H**) Knockdown of *syx-17* or *vamp-7* does not alter the lifespan shortening observed upon *uso-1* RNAi-silencing. Concomitant knockdown of *syx-17* and *vamp-7* results in a reduction in lifespan while single knockdown does not affect longevity of otherwise wild-type animals. (* *p* < 0.05, ** *p* < 0.01, *** *p* < 0.001, **** *p* < 0.0001, ns denotes non-significant difference, Mantel-Cox test). Lifespan values are provided in Supplementary Table S1.

Neurotransmitters are considerably altered in normal aging and age-related neurodegenerative diseases, however, the role of neurosecretory SNAREs or even of mitoSNARE proteins in aging has not been appreciated yet [53,54,55]. To directly examine whether mitoSNAREs regulate organismal aging, we inhibited *syx-17*, *vamp-7*, *snb-6, uso-1* or *nsf-1* selectively in neurons, and survival rates were monitored. Intriguingly, neuron-specific inhibition of *syx-17*, *vamp-7*, *snb-6, uso-1* and *nsf-1* significantly reduced the lifespan of otherwise wild-type animals (Figure 5A–D and Figure S5A). In contrast, *syx-17*, *vamp-7* or *snb-6* knockdown in all other tissues except neurons, did not have any effect on the lifespan (Figure 5E). Interestingly, we found that simultaneous inhibition of *syx-17* and *vamp-7* in all tissues caused a significant reduction in lifespan (Figure 5F), consistent with the idea that SYX-17 and VAMP-7 act synergistically to regulate mitochondrial maintenance. We propose that single depletion of either SYX-17 or VAMP-7 in non-neuronal tissues is likely tolerated as they may exhibit functional redundancy with other SNARE members, while its simultaneous depletion is detrimental to lifespan, as it blocks the SNARE-dependent pathway of mitochondrial maintenance (Figure 5F).

As previously shown, STX17 regulates mitochondria fission via the control of DRP1 mobility and activity [47]. In line with this, we documented that while the single inhibition of either *syx-17* or *drp-1* did not affect lifespan, concomitant loss of DRP-1 and SYX-17 reduced lifespan significantly (Figure S5B). Our findings provide evidence that DRP-1 and SYX-17 functionally interact to regulate longevity in *C. elegans*.

Finally, we observed a striking reduction in lifespan upon inhibition of either *uso-1* or *nsf-1* in all tissues tested, suggesting that SNARE tethering and disassembly factors are broadly needed for normal aging (Figure 5D,E, and Figure S5A). In addition, loss of SYX-17 or VAMP-7 failed to ameliorate the lifespan-shortening effects of USO-1-deficient animals, suggesting that USO-1 acts downstream of SYX-17 and VAMP-7 to exert its effect on lifespan (Figure 5G,H).

## 3. Discussion

SNARE proteins were originally identified as essential components of the brain synaptic vesicles, and since then, their role in vesicle fusion and trafficking has been well-appreciated [5,56]. Recently, the contribution of SNARE fusogenic activity to autophagosome-lysosome fusion has also received great attention [13,16,57,58,59]. In this study, we report a previously unrecognized subset of SNARE proteins that localize to mitochondria, and present findings indicating their contribution to basal autophagy regulation and aging.

As previously shown in mammals, the autophagosomal t-SNARE, syntaxin 17 (STX17) and its cognate v-SNAREs, lysosomal VAMP7 or VAMP-8 as well as synaptosomal-associated protein 29 (SNAP29) come together upon nutrient stress to overcome the energy barrier of autolysosome membrane bridging [17]. Later studies have also shown that the repertoire of STX17/SYX-17 functions embraces the regulation of autophagy initiation/maturation and mitochondrial quality control mechanisms including mitophagy, mitochondrial dynamics and MDVs. In this regard, the biogenesis of autophagosomes at the mitochondrial-associated membrane (MAM) relies on STX17 binding to ATG14 and its recruitment from the ER to the MAM upon nutrient starvation [60]. STX17 is also present in the Golgi apparatus and upon starvation is phosphorylated by TBK1 at S202, and in concert with autophagy initiation components such as FIP200, ATG13 and ULK1, triggers autophagy formation [61]. Furthermore, STX17 controls mitochondrial fission by regulating DRP1 (dynamin-related protein 1) phosphorylation state in a RAB32-PKA-dependent manner [47]. Under stress and starvation, STX17 also regulates PGAM5 (a mitochondrial protein phosphatase) localization and interaction with FUNDC1, a well-recognized mitophagy receptor [46]. Due to SNARE pairing specificity among v-/t-SNAREs and SNARE-coupling proteins, we hypothesized that additional mitochondrial SNAREs exist and exert similar and synergistic functions.

In this regard, we initially predicted that a subset of SNARE proteins is exported to mitochondria (Figure S1A–C). Interestingly, we experimentally verified the mitochondrial presence of SYX-17, VAMP-7, SNB-6 and USO-1 in *C. elegans* (Figure 1C and Figure S2C). Paradoxically, while the nematode SYX-17 heterologously expressed in HeLa cells, was present in mitochondria, it was neither colocalized with LC3 dots nor could it compensate for the absence of human STX17 regarding autophasome formation upon starvation [39]. This discrepancy could be due to the different physiology of this specific cancer cell line or to the lack of its cognate assembly and disassembly factors and needs to be further explained. In the present study, we verified the conserved role of SYX-17 in mitochondrial abundance and autophagy regulation, and we present unrecognized interactors of SYX-17 such as the SNARE tethering and disassembly factors, USO-1 and NSF-1. Interestingly, the effect of mitoSNARE assembly proteins, SYX-17, VAMP-7 and SNB-6 on mitochondrial mass and autophagy heavily rely on the activity of the SNARE disassembly factor, NSF-1 (Figure 2F,G, and Figure 4C). In response to oxidative stress, mammalian STX17 also decorates mitochondrial-derived vesicles (MDVs) and drives MDV-endolysosomal fusions through its interaction with SNAP29-VAMP7 [31]. Strikingly, HeLa cells treated with antimycin A showed increased formation of MDVs, which were positive for matrix-derived materials and negative for outer mitochondrial membrane (OMM) material, as they were TOM20-negative vesicles [45]. It was shown that VAMP7-positive vesicles did not incorporate OMM-related TOM20 [45]. In contrast, we provide evidence that VAMP-7-positive MDVs can also transfer OMM-related material as they largely co-localize with TOMM-20-positive vesicles in body wall muscle cells (Figure 1D). Additional studies on the mitochondrial role of SNARE assembly and disassembly proteins, SYX-17, VAMP-7, SNB-5/6/7, USO-1 and NSF-1 identified here, will further decipher the specificity of the trafficking mechanisms employed by MDVs.

Despite the fact that SNAREs participate in fundamental cellular processes pertinent to aging and age-associated disorders, their role in lifespan control has just begun to be elucidated. Early studies in *C. elegans* have shown that loss of syntaxin *unc-64*, the human ortholog of STX1, promotes longevity through the insulin/IGF1 signaling pathway [62]. Interestingly, mice overexpressing syntaxin 4 (STX4) lived about 33% more than the wild-type counterparts whilst insulin secretion and sensitivity were improved [63]. A recent report also shows that mitochondrial STX4 regulates DRP1 phosphorylation at S637, which favors mitochondrial fusion [24]. Our observations point out the detrimental effect of mitoSNARE inhibition on normal aging specifically in neurons (Figure 5A–D and Figure S5A). In non-neuronal tissues, we noticed that NSF-1 and USO-1 are critical determinants of the aging process, while simultaneous depletion of SYX-17 and VAMP-7 is also detrimental to normal lifespan (Figure 5F–H). Since mitochondrial control of aging is an active field of research, we expect future studies to shed light on mitoSNARE protein contribution to the aging process.

In the present study, we identified previously unrecognized mitochondrial SNAREs and reported their synergistic roles in mitochondrial abundance and morphology, basal autophagy regulation and aging. Additional effort is required to fully understand SNARE assembly and disassembly proteins, redundant and complementary actions in both autophagy and aging.

## 4. Materials and Methods

### 4.1. Strains

We followed standard procedures for *C. elegans* maintenance and other genetic manipulations [64]. Nematodes were grown at 20 °C unless noted otherwise. The following strains, available at Caenorhabditis Genetics Center (CGC), were used: N2: Wild type Bristol isolate, DA2123: *adIs2122* [lgg-1p::GFP::lgg-1 + *rol-6(su1006*)]), HZ589: *bpIs151*[sqst-1p::sqst-1::GFP + *unc-76(+)*], TU3401: sid-1(pk3321) V; *uIs69* V, JJ2586: cox4(*zu476*[cox-4::eGFP::*3xFLAG*]) I, RW12185: atg-4.1(*st12185*[atg-4.1::TY1::EGFP::*3xFLAG*]), PS6187: *syEx1155* [myo-3p::tomm-20::mRFP::*3xMyc* + *Cbr-unc-119(+)*]. Strains produced in this study by micro-injections [*rol-6(su1006*)] or microparticle bombardment [*Cbr-unc-119(+)*]: N2; *Ex*[psyx-17::SYX-17::mCherry;pRF4], PS6187: *syEx1155* [myo-3p::tomm-20::mRFP::*3xMyc* + *Cbr-unc-119(+)*] + *Ex*[pmyo-3::VAMP-7::GFP;pRF4], DA2123: *adIs2122* [lgg-1p::GFP::lgg-1 + *rol-6(su1006*)] + *Ex*[psyx-17::SYX-17::mCherry;*pRF4*], N2; *Ex*[puso-1::USO-1::GFP;*pRF4*], N2;*Ex*[psyx-17::SYX-17::mCherry + puso-1::USO-1::GFP;*pRF4*], N2;*Ex*[plgg-1::DsRed::LGG-1 + puso-1::USO-1::GFP;*pRF4*], *unc-119 (ed 3) III*; *Ex*[puso-1::USO-1::GFP+*cb-unc-119(+)*], *unc-119 (ed 3) III*; *Ex*[pmyo-3::SNB-6::dsRed+*cb-unc-119(+)*]. We noted that overexpression of *snb-6* and *vamp-7* by their endogenous promoters could not be tolerated by the animals, as we obtained F1 transgenics that were not able to produce viable progeny, thus the line could not be sustained. For *syx-17* and *uso-1*, we obtained transgenics with expression under the endogenous promoter, however, their expression levels were low. Moreover, when two transgenes were co-injected, oftentimes they were not expressed in the same cell/tissue. Tissue-specific expression of mitoSNARE components in body wall muscles was better tolerated.

### 4.2. Feeding RNAi

Feeding RNAi was performed as previously described [65]. All RNAi treatments were started upon hatching and continued lifelong or until the indicated day of measurement. RNAi of single genes was performed by cloning the coding regions of interest within the two T7 RNA polymerase promoters. Simultaneous RNAi knockdown of more than one gene was performed by mixing different dsRNA expressing bacterial cultures of similar ODs in a 1/1 ratio. Single RNAi treatments in these experiments were performed by mixing the culture of the desired dsRNAi expressing bacteria with the bacteria bearing the empty vector (pL4440) (Control).

### 4.3. Molecular Cloning

The cloning strategy for all RNAi constructs entails PCR amplification of the gene of interest from the nematode genomic DNA or cDNA followed by cloning of each PCR fragment into pCR-II TOPO vector (Invitrogen-Thermo Fisher Scientific, Waltham, MA, USA). cDNA was synthesized using the PrimeScript Reverse Transferase kit (2680A, Takara, TAKARA BIO INC, Shiga, Japan). Proper restriction enzymes were used to subclone genes from the pCR-II TOPO vector into the pL4440 vector for T7-dependent expression of dsRNA. For *syx-17*, a PCR fragment of ~1900bp using genomic DNA as a template was cloned into pL4440 using BamHI/NotI restriction enzymes. For *uso-1*, a PCR fragment of ~2500bp, using cDNA as a template, was cloned into pL4440 using KpnI/XbaI restriction enzymes. For *nsf-1*, a PCR fragment of ~5500bp, using genomic DNA as a template, was cloned into pL4440 using BamHI/KpnI restriction enzymes. RNAi clones of *vamp-7* and *snb-6* were obtained from Ahringer’s RNAi library (Source Biosciences, Cambridge, UK). For the construction of the SYX-17 translational reporter, we PCR amplified the promoter region of *syx-17* (~2000bp) and cloned into pPD95.77 plasmid vector carrying mCherry. Next, the entire coding region of *syx-17* was cloned into the final vector using SmaI/AgeI restriction enzymes. For the construction of the USO-1 translational reporter, we PCR amplified the promoter region of *uso-1* (~700bp) and cloned into pPD95.77 plasmid vector carrying GFP using XbaI/PstI restriction enzymes. Then, cDNA of *uso-1* (~2800bp) was cloned into the final vector using PstI/AgeI restriction enzymes. For the construction of the VAMP-7 translational reporter, the cDNA region of *vamp-7* was PCR amplified and cloned into the pPD95.77 vector (expressing GFP under *myo-3* promoter) using BamHI/AgeI restriction enzymes. Similarly, for the construction of the SNB-6 translational reporter, we PCR amplified the coding region of *snb-6* and cloned into the pPD95.77 vector (expressing GFP under myo-3 promoter) using BamHI/AgeI restriction enzymes. Primers used for the amplification of specific ORFs are provided in Supplementary Table S4.

### 4.4. Prediction of Mitochondrial Localization

To predict subcellular localization and mitochondrial targeting signals between SNARE proteins we analyzed protein sequences retrieved from WormBase using DeepLoc 2.0(DTU Health Tech, Lyngby, Denmark) (https://services.healthtech.dtu.dk/service.php?DeepLoc-2.0) (accessed on 16 July 2022), MitoProt II (Institute of Human Genetics, Munich, Germany) (https://ihg.helmholtz-muenchen.de/ihg/mitoprot.html) (accessed on 29 July 2022) and iMLP Technical University of Kaiserslautern, Kaiserslautern, Germany) (http://imlp.bio.uni-kl.de/) (accessed on 26 August 2022) tools [35,36,37].

### 4.5. Phylogenetic Analysis of SNARE Proteins

For this study, about 30 *C. elegans* SNARE and SNARE-associated protein sequences were retrieved directly from WormBase based on previous annotation (https://wormbase.org) (accessed on 11 May 2022) [28]. The protein sequences of their closest human orthologs were retrieved from UniProt (https://www.uniprot.org) (accessed on 11 May 2022). Initially, the protein sequences obtained were aligned using Clustal Omega, which also gave a neighbor-joining tree without distance corrections (https://www.ebi.ac.uk/Tools/msa/clustalo/) (accessed on 12 May 2022) [66]. The phylogenetic tree data obtained from Clustal Omega were then further annotated using iTOL (https://itol.embl.de) (accessed on 8 August 2022). Particularly, the unrooted display mode was selected using the equal-angle algorithm by default [29].

### 4.6. Immunoblotting

Synchronous animal populations under all conditions tested were harvested and used for whole-worm lysate preparation. For total worm protein extraction, animals were collected and washed in M9 buffer. After washing, 2 volumes of homogenization buffer (20 mM Tris, pH 7,4, 20 mM NaCl, 1 mM MgCl_2_) plus a complete mini proteinase inhibitor cocktail (Roche) was added in the worm pellets together with 1 volume of beads (0.5 mm Zirconium Oxide Beads). Samples were placed in the Bullet Blender Homogenizer (Model BT24M, Next Advance, Troy, NY, USA) for 3 min at Speed 10. After homogenization 6x, Laemmli sample buffer with b-mercaptoethanol was supplemented in a final concentration 1x. Samples were boiled for 5 min at 95 °C. Protein samples were analyzed by 12% Tricine-SDS–polyacrylamide gel electrophoresis (SDS–PAGE), transferred on nitrocellulose membrane, and blotted against various antibodies. The antibodies used for this study were anti-alpha-tubulin (DSHB, AA4.3 (concentrate)) used in dilution 1/2000, anti-atp5A (Abcam, ab14748), used in dilution 1/1000 and anti-GFP (Minotech), used in dilution 1/2000.

### 4.7. Lifespan Assays

Lifespan assays were performed at 20 °C. Synchronous animal populations were generated by hypochlorite treatment of gravid adults. For RNAi lifespan experiments, worms were placed on NGM plates seeded with HT115 (DE3) bacteria transformed with either the pL4440 vector or the test RNAi construct. IPTG in a final concentration of 2 mM was also added for RNAi induction. At the L4-young adult larval stage animals were transferred to fresh plates in groups of around 30 worms per plate. Animals were transferred to fresh plates every 2 days thereafter and examined every day for touch-provoked movement and pharyngeal pumping, until death. We counted as “censored” worms that died owing to internally hatched eggs, extruded gonad or desiccation due to crawling on the edge of the plates. Each survival assay was repeated at least two times. Survival curves were created using the product-limit method of Kaplan and Meier. For statistical analysis of the survival curves, we used the log-rank (Mantel–Cox) test. Statistics and lifespan curve generation was accomplished by using the Prism software package (GraphPad Prism 8 Software). Full lifespan statistics are summarized in Supplementary Tables S1 and S2.

### 4.8. Detection of Autophagy

To measure the number of autophagosomes in vivo we used the DA2123 (*adIs2122* [lgg-1p::GFP::lgg-1 + rol-6(su1006)]) strain. We quantified the GFP positive puncta in the seam cells of adult day 1 animals using the 60x lens of a widefield epifluorescence microscope (EVOS Cell Imaging Systems, Thermo Fisher Scientific, Waltham, MA, USA). Additionally, protein from whole animal extracts was used to quantify the levels of unlipidated and lipidated LGG-1 (the C. elegans ortholog of LC3/Atg8) under the tested conditions. Additionally, we calculated SQST-1::GFP (the *C. elegans* ortholog of SQSTM1) positive puncta using the HZ589: *bpIs151*[sqst-1p::sqst-1::GFP + *unc-76(+*)] strain. Puncta were measured in the head region or in the seam cells of the animals. Fluorescent images were acquired using the EVOS Cell Imaging Systems (Thermo Fisher Scientific, Waltham, MA, USA). For the assessment of autophagy flux, we used the DLM1 reporter strain bearing the transgene (eft-3p::CERULEAN-VENUS::lgg-1 + unc-119(+)). Processing of dual fluorescent protein fused to LGG-1 (dFP::LGG-1) upon fusion with the lysosome led to the production of monomeric fluorescent protein (mFP), detected by anti-GFP western blot.

### 4.9. Mitotracker Staining

For staining of the animals with Mitotracker Red CM-H2X ROS (Molecular Probes, Invitrogen, M7513, Thermo Fisher Scientific, Waltham, MA, USA) and Mitotracker Green FM (Molecular Probes, Invitrogen, M7514, Thermo Fisher Scientific, Waltham, MA, USA), we diluted the stain in M9 buffer and placed it at the top of pre-seeded plates in a final concentration of 0.28 μM. We let the worms feed overnight, and the next day, we measured total mtROS levels under the tested conditions.

### 4.10. Microparticle Bombardment

The Biolistic PDS-1000/He particle delivery system (BioRad, Hercules, CA, USA) was used for biolistic transformation. For generation of transgenic strains we linearized 10–15 μg plasmid DNA, which was bombarded onto unc-119(ed3) mutant L4/adult hermaphrodites. Animals were grown on NGM plates seeded with Na22 bacteria and the rest of the procedure was done as previously described [67].

### 4.11. RNA Isolation and qPCR Analysis

Total mRNA was isolated from synchronized day 1 adults, which were lysed in 250 μL of TRIzol by freeze-cracking (Invitrogen-Thermo Fisher Scientific, Waltham, MA, USA). For cDNA synthesis, mRNA was reverse transcribed using iScriptTM cDNA Synthesis Kit (BioRad, Hercules, CA, USA). Quantitative PCR was performed in triplicate using a BioRad CFX96 Real-Time PCR system (BioRad, Hercules, CA, USA). Expression levels of the housekeeping genes *act-3* and/or *pmp-3* were used as an internal control for normalization. Unless stated otherwise, at least three independent experiments were performed for each condition. Real-time PCR primers are provided in Supplementary Table S4.

### 4.12. Quantification and Statistical Analysis

Quantification was performed using the Image J software (NIH, Bethesda, MD, USA) (http://imagej.nih.gov/ij/) (accessed multiple times) as specified in the Figure legends. Fluorescent market colocalization was assessed using the BIOP JACoP plugin (https://c4science.ch/w/bioimaging_and_optics_platform_biop/image-processing/imagej_tools/jacop_b/) (accessed on 23 January 2023) in areas of the worms where both markers were expressed. We measured Manders Overlap Coefficient and Manders Fractional Colocalization Coefficients M1 (for the red channel) and M2 (for the green channel) [68] (Supplementary Table S3). Statistical analyses were carried out using the Prism software package (GraphPad Software Inc., San Diego, CA, USA) and the Microsoft Office 2010 Excel software package (Microsoft Corporation, Redmond, WA, USA). The statistical tests applied for each experiment are specified in the Figure legends.

## Data Availability

Data relative to the findings of this study are available from the corresponding author upon request.

## Supplementary Materials

The following supporting information can be downloaded at: https://www.mdpi.com/article/10.3390/ijms24044230/s1.
