# Supplementary material for: MitoSNARE Assembly and Disassembly Factors Regulate Basal Autophagy and Aging in C. elegans
Source: Int J Mol Sci. 2023 Feb 20;24(4):4230. doi: 10.3390/ijms24044230 (PMC9964399; doi:10.3390/ijms24044230)
Supplement: Supplementary file 1 [file ijms-24-04230-s001.zip › ijms-2165363-supplementary.pdf]

MitoSNARE assembly and disassembly factors regulate basal autophagy and aging in *C. elegans*

Ilias Gkikas[1,2†], Ioanna Daskalaki[1,2,†], Konstantinos Kounakis[3,1], Nektarios Tavernarakis[1,3*] & Eirini Lionaki[1*]

[1]Institute of Molecular Biology and Biotechnology, Foundation for Research and Technology - Hellas,

[2]Department of Biology, School of Sciences and Engineering, University of Crete

[3]Department of Basic Sciences, Faculty of Medicine, University of Crete, Heraklion 71110, Crete, Greece

Supplementary Materials

Contents

Supplementary Figure S1

Supplementary Figure S2

Supplementary Figure S3

Supplementary Figure S4

Supplementary Figure S5

Supplementary Figure S6

Supplementary Table S1

Supplementary Table S2

Supplementary Table S3

Supplementary Table S4

A

DeepLoc 2: prediction of subcellular localization-associated sorting signals

SYX-17: two transmembrane domains, **Probability**: 0.712

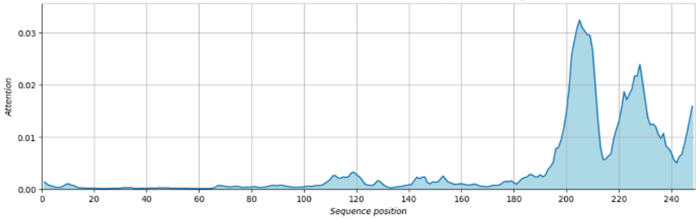

SNB-5: one transmembrane domain, **Probability**: 0.554

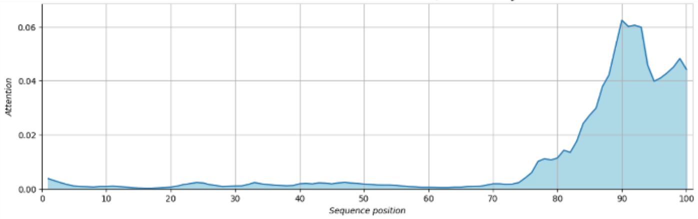

SNB-6: one transmembrane domain, **Probability**: 0.684

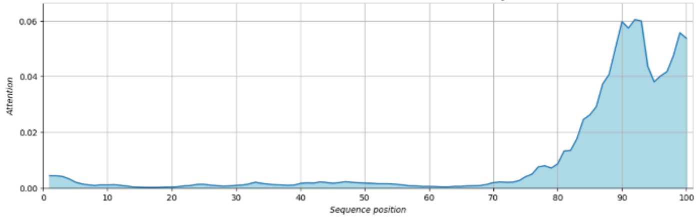

SNB-7: one transmembrane domain, **Probability**: 0.546

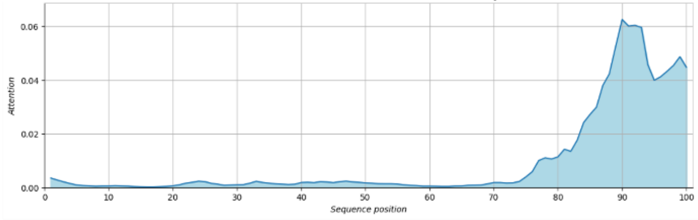

B

MitoProt II: prediction of N-terminal matrix-targeting signal (MTS)

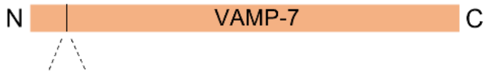

Cleavage site 14
Cleavage sequence: MPGRVMSVVLCRP
Probability of export to mitochondria: 0.0484

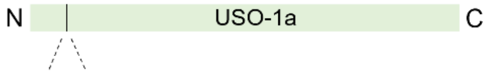

Cleavage site 67
Cleavage sequence: MFAYSTSSITGSTLSVRSFSSDTTISNAFFNLPQSNFRQRLQAKKRSPKRVSKRAPLRTHPPIPRQ
Probability of export to mitochondria: 0,9615

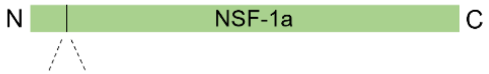

Cleavage site 24
Cleavage sequence: MSPVPCLSTCCCFRKIVEYSTMS
Probability of export to mitochondria: 0,6892

C

iMLP:: prediction of internal MTS-like signals (iMTS-Ls)

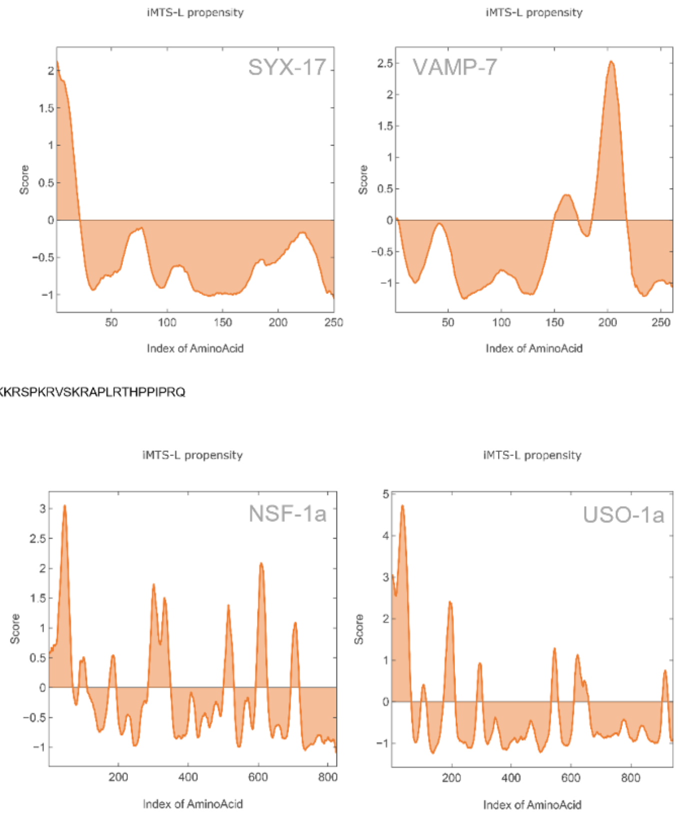

Supplementary Figure S1

Supplementary Figure S1. Analysis of about 30 SNARE and SNARE-coupling protein sequences for sub-cellular localization.

(A) Protein sequences analysis with DeepLoc2.0 reveal 4 mitochondrial SNARE proteins (B) MitoProtII prediction revealed the mitochondrial targeting signal of VAMP-7, USO-1 and NSF-1 alongside the cleavage sites at the N-terminal. (C) iMLP prediction showing the presence of internal targeting signal in SYX-17, VAMP-7, NSF-1 and USO-1.

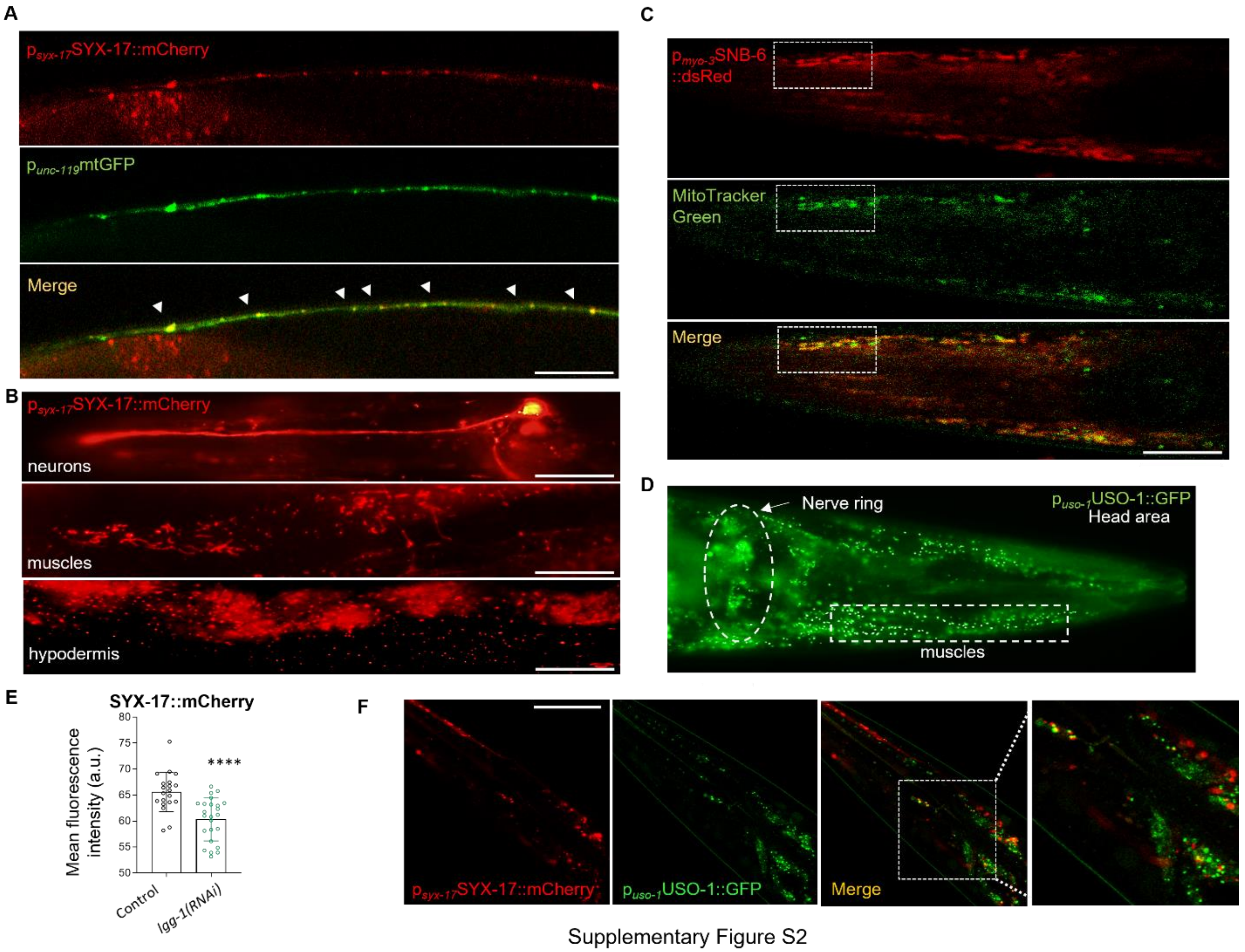

Supplementary Figure S2

Supplementary Figure S2. Expression pattern of mitoSNAREs in neuronal and non-neuronal tissues.

(A) Transgenic animals expressing SYX-17 fused with mCherry and mitochondrial targeted GFP paneuronally driven by *unc-119* promoter (63x lens, scale bar: 150μm). (B) Different expression pattern of SYX-17::mCherry in various tissues including neuronal, muscle and hypodermal cells (40x lens, scale bar: 100μm). (C) Transgenic animals expressing SNB-6 fused with dsRed in muscle cells and mitochondria stained with MitoTracker Green (40x lens, scale bar: 50μm). (D) Transgenic animals expressing USO-1 fused with GFP under the control of its own promoter depicting its doted expression pattern in body-wall muscles, head region muscle and neuronal cells (40x lens, scale bar: 100μm). (E) Protein levels of SYX-17::mCherry expressing animals upon depletion of LGG-1 (****p<0.0001 unpaired two-tailed t-test). (F) Transgenic animals expressing SYX-17 fused with mCherry and USO-1 fused with GFP. Both reporters are driven by their endogenous promoter. (63x lens, scale bar: 150μm) (MOC= 0,9040 ± 0,01532, $M1_{(SYX-17)}$= 0,1880 ± 0,04467, $M2_{(USO-1)}$= 0,2985 ± 0,06687).

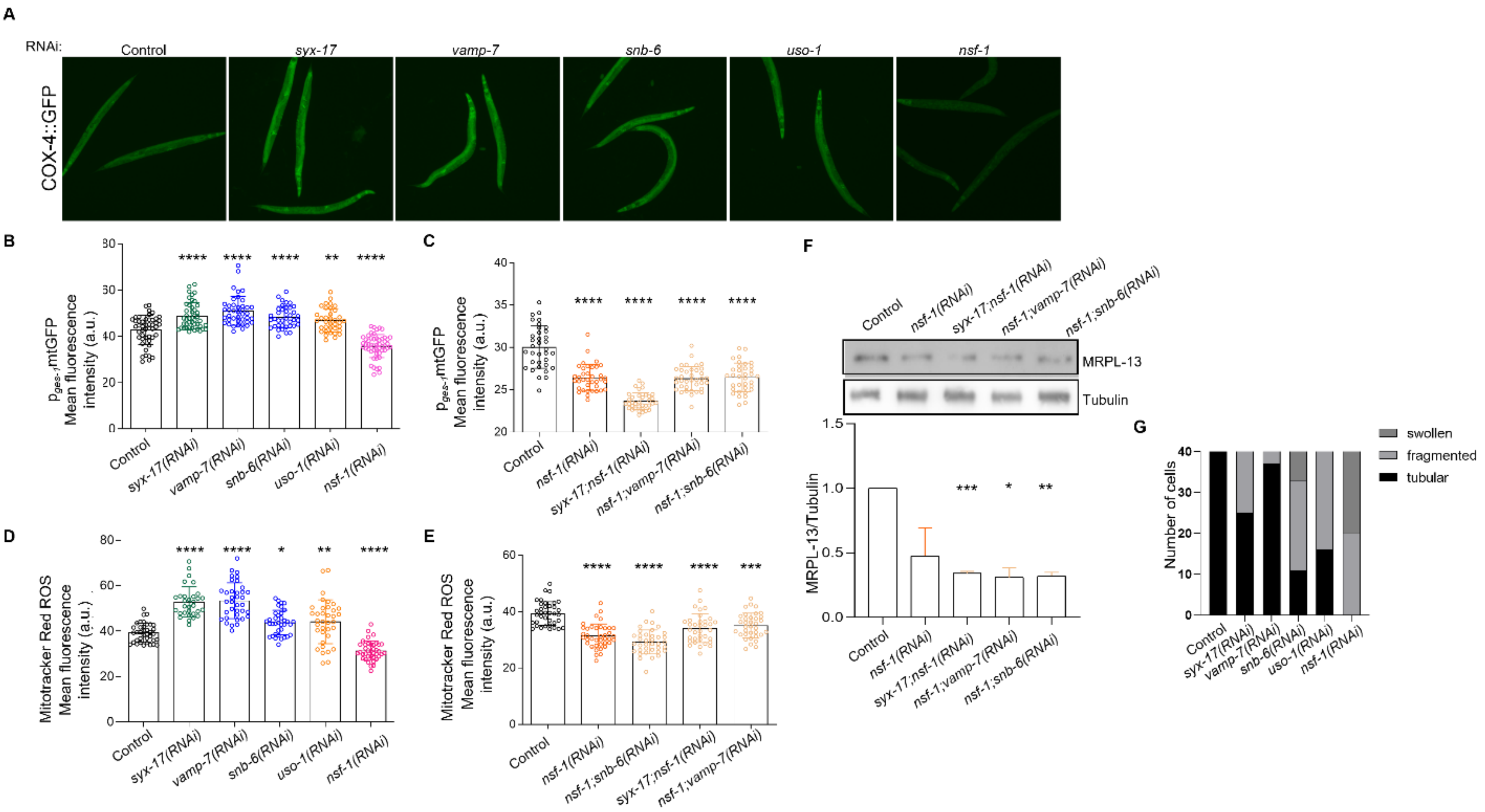

Supplementary Figure S3

Supplementary Figure S3. RNAi depletion of mitoSNAREs affects mitochondrial mass.

(A) Representative images of the single copy COX-4::GFP reporter strain treated with the indicated RNAi bacterial clones. (B) Quantified relative fluorescence of the $P_{ges\text{-}1}$mtGFP reporter strain treated with the indicated RNAi bacterial clones. (n=259, **p=0.0035, **** p<0.0001, one-way ANOVA). The experiment was performed in 3 biological independent replicates with similar results. (C) Quantified relative fluorescence of the $P_{ges\text{-}1}$mtGFP reporter strain treated with the indicated RNAi bacterial clones. (n=180, **** p<0.0001, one-way ANOVA). The experiment was performed in 3 biological independent replicates with similar results. (D) Quantified relative fluorescence of wild type worms treated with the indicated RNAi bacterial clones and subsequently stained with the mitochondrial ROS-specific dye Mitotracker Red CM-H2X ROS. (n=215, *p=0.0178, **p=0.0089, **** p<0.0001, one-way ANOVA). The experiment was performed in 2 biological independent replicates with similar results. (E) Quantified relative fluorescence of wild type worms treated with the indicated RNAi bacterial clones and subsequently stained with the mitochondrial ROS-specific dye Mitotracker Red CM-H2X ROS. (n=183, ***p=0.0003, **** p<0.0001, one-way ANOVA). The experiment was performed in 2 biological independent replicates with similar results. (F) Immunoblot analysis of endogenous MRPL-13 protein levels of adult day 1 animals fed with the indicated dsRNA expressing bacteria (upper panel) and the respective quantification of the normalized protein levels (lower panel). n = 2 biologically independent experiments (*p=0.0114, **p=0.0017, *** p=0.0006, unpaired two-tailed t-test). (G) Quantification of mitochondrial network phenotypes observed upon the indicated RNAi conditions.

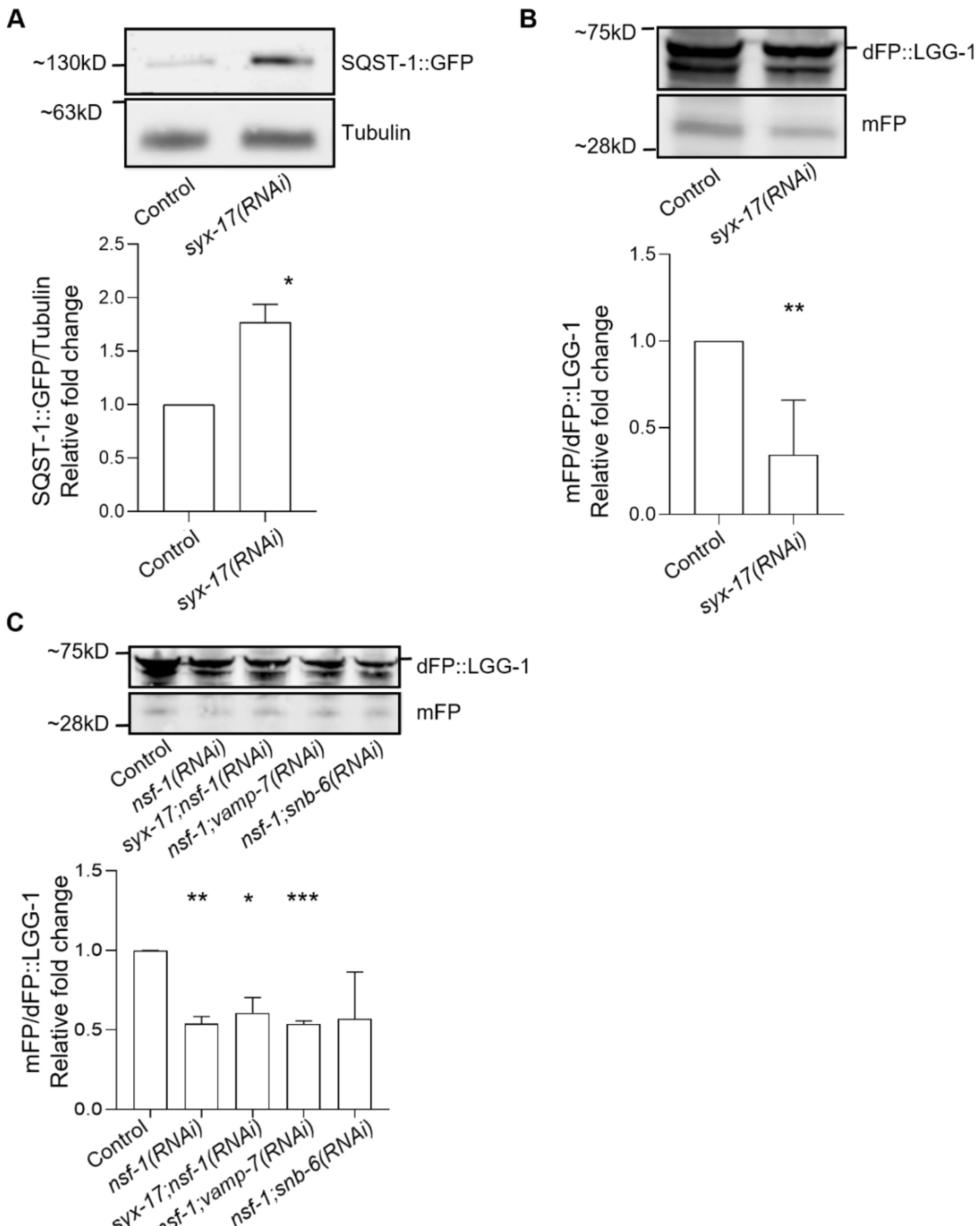

Supplementary Figure S4

Supplementary Figure S4. mitoSNARE depletion blocks basal autophagic flux

(A) Immunoblot analysis of total SQST-1::GFP protein levels of adult day 1 animals fed with the indicated dsRNA expressing bacteria (upper panel) and the respective quantification of the normalized protein levels (lower panel). n = 2 biologically independent experiments (*p= 0.0451, unpaired two-tailed t-test). (B) Immunoblot analysis of dual fluorescent protein (dFP) fused with LGG-1 and monomeric fluorescent protein (mFP) protein levels of adult day 1 animals, fed with the indicated dsRNA expressing bacteria (upper panel) and the respective quantification of the normalized ration of mFP to dFP::LGG-1 protein levels (lower panel). n = 4 biologically independent experiments (**p=0.0060, unpaired two-tailed t-test). (C)) Immunoblot analysis of dual fluorescent protein (dFP) fused with LGG-1 and monomeric fluorescent protein (mFP) protein levels of adult day 1 animals, fed with the indicated dsRNA expressing bacteria (upper panel) and the respective quantification of the normalized ration of mFP to dFP::LGG-1 protein levels (lower panel). n = 2 biologically independent experiments (*p=0.0297, **p=0.0045, ***p=0,0010, unpaired two-tailed t-test).

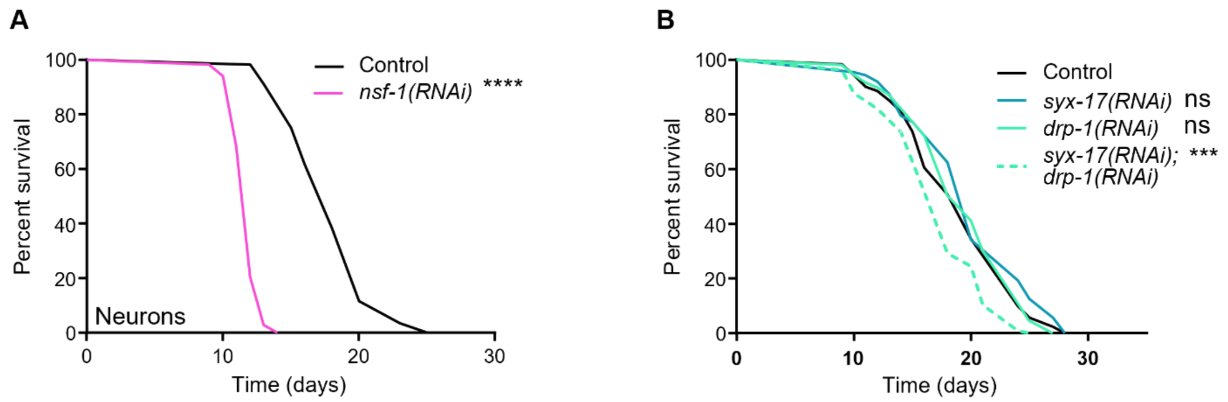

Supplementary Figure S5. mitoSNAREs regulate normal aging

(A) Neuronal-specific inhibition of *nsf-1* dramatically shortens the lifespan of otherwise wild-type animals. (B) Concomitant inhibition of *syx-17* and *drp-1* results in reduced lifespan while single inhibition does not alter the animal's lifespan. Lifespan values are provided in Supplementary Table S2.

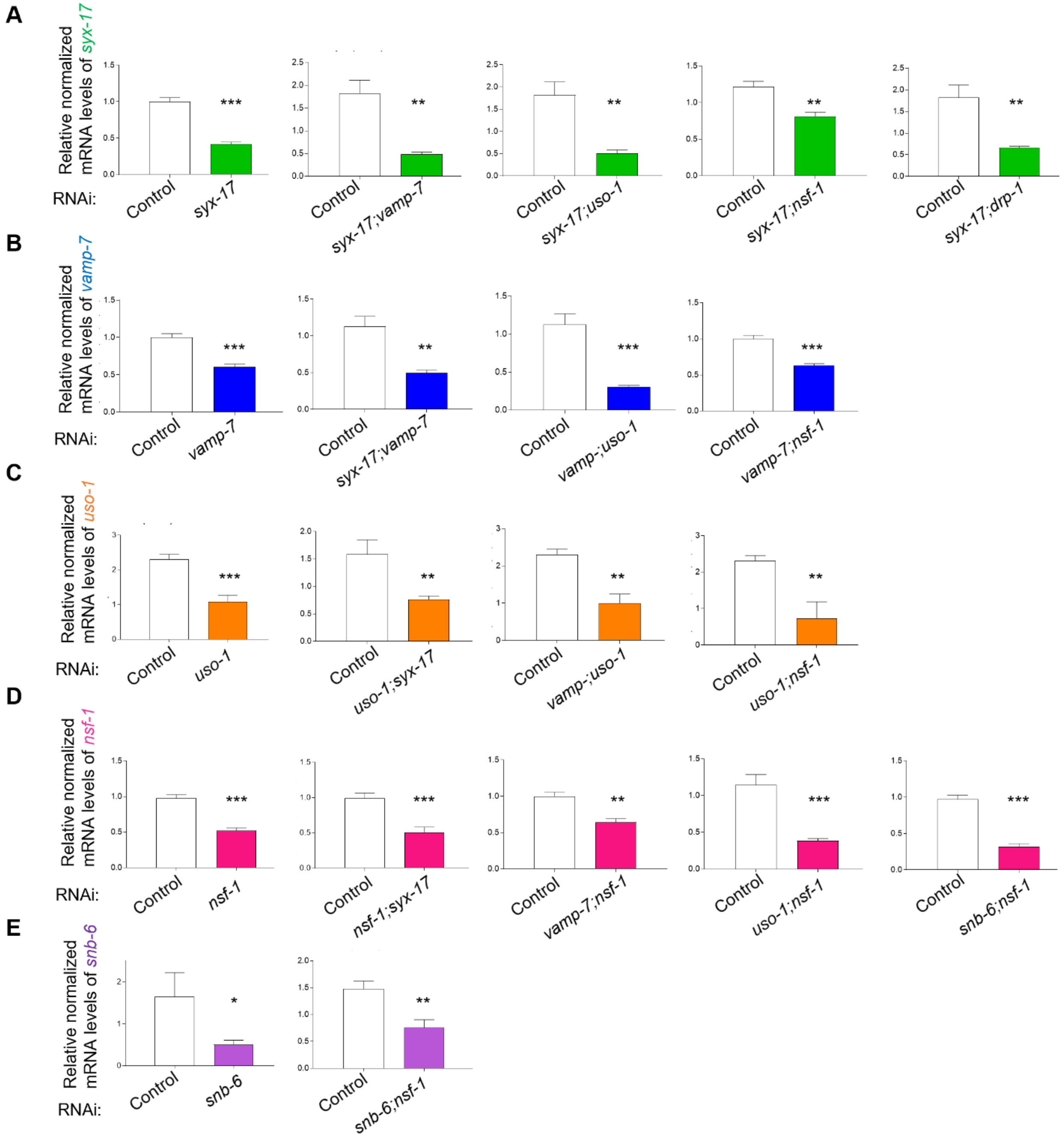

Supplementary Figure S6. mRNA levels upon RNAi depletion of the indicated genes.

mRNA levels of (A) *syx-17*, (B) *vamp*-7, (C) *uso*-1, (D) *nsf-1*, (E) *snb*-6 gene upon single or double RNAi depletion with the indicated bacterial clones. Expression was normalized with the expression levels of the housekeeping gene *pmp-3.* (*p<0.0332, **p<0.0021, ***p<0.0002, unpaired two-tailed t-test)

Supplementary Table S1. Lifespan data/Statistics

Stain name	Treatment	No of deaths	Median lifespan	Significance, P value (compared to control RNAi)
TU3401	control RNAi	106	18	
	syx-17(RNAi)	121	16	**, 0.0023
TU3401	control RNAi	98	18	
	syx-17(RNAi)	145	17	****, <0.0001
TU3401	control RNAi	124	18	
	syx-17(RNAi)	159	16	****, <0.0001
TU3401	control RNAi	103	18	
	vamp-7(RNAi)	77	15	****, <0.0001
TU3401	control RNAi	88	18	
	vamp-7(RNAi)	102	17	**, 0.0078
TU3401	control RNAi	96	17	
	snb-6(RNAi)	99	14	*, 0.0117
TU3401	control RNAi	79	19	
	snb-6(RNAi)	85	16	****, <0.0001
TU3401	control RNAi	112	18	
	uso-1(RNAi)	127	16	***, 0.0001
TU3401	control RNAi	80	17	
	uso-1(RNAi)	86	15	***, 0.0002
N2	*control RNAi*	132	20	
	syx-17(RNAi)	96	20	ns, 0.1147
	vamp-7(RNAi)	103	20	ns, 0.8335
	snb-6(RNAi)	107	20	ns, 0.6578
	uso-1(RNAi)	230	18	****, <0.0001
	nsf-1(RNAi)	248	12	****, <0.0001
N2	*control RNAi*	93	20	
	syx-17(RNAi)	113	20	ns, 0.1135
	vamp-7(RNAi)	78	20	ns, 0.0506
	snb-6(RNAi)	93	20	ns, 0.0874
	uso-1(RNAi)	113	16	*, 0.0259
	nsf-1(RNAi)	79	12	****, <0.0001
N2	control RNAi	137	19	
	syx-17(RNAi)	176	19	ns, 0.2963
	vamp-7(RNAi)	189	19	ns, 0.1281
	syx-17;vamp-7(RNAi)	194	20	****, <0.0001
N2	control RNAi	104	20	
	syx-17(RNAi)	106	20	ns, 0.7598
	vamp-7(RNAi)	102	20	ns, 0.5181
	syx-17;vamp-7(RNAi)	81	18	**, 0.009
N2	*Control RNAi*	128	22	
	uso-1(RNAi)	238	19	****, <0.0001
	syx-17(RNAi)	146	21	ns, 0.6395
	syx-17;uso-1(RNAi)	152	18	****, <0.0001
N2	*control RNAi*	101	20	
	uso-1(RNAi)	96	18	****, <0.0001
	syx-17(RNAi)	110	20	ns, 0.1404
	syx-17;uso-1(RNAi)	148	18	****, <0.0001
N2	*control RNAi*	122	21	
	uso-1(RNAi)	225	18	****, <0.0001

	vamp-7(RNAi)	162	20	ns, 0.6307
	uso-1;vamp-7(RNAi)	208	19	****, <0.0001
N2	*control RNAi*	117	20	
	uso-1(RNAi)	80	18	****, <0.0001
	vamp-7(RNAi)	145	20	ns, 0.3240
	uso-1;vamp-7(RNAi)	100	16	****, <0.0001

Supplementary Table S2: Lifespan data/Statistics

Strain name	Treatment	No of deaths	Median lifespan	Significance, P value (compared to control RNAi)
TU3401	control RNAi	112	18	
	nsf-1(RNAi)	284	12	****, <0.0001
TU3401	control RNAi	91	17	
	nsf-1(RNAi)	65	14	****, <0.0001
N2	control RNAi	122	20	
	syx-17(RNAi)	88	20	ns, 0.1088
	drp-1(RNAi)	109	20	ns, 0.9416
	syx-17;drp-1(RNAi)	106	18	***, <0.0002
N2	control RNAi	103	21	
	syx-17(RNAi)	99	21	ns, 0.0734
	drp-1(RNAi)	100	21	ns, 0.4209
	syx-17;drp-1(RNAi)	93	18	****, <0.0001

Supplementary Table S3. Values of Manders Overlap Coefficient (MOC) and Manders Fractional Colocalization Coefficients M1 (for the red channel) and M2 (for the green channel) for the indicated experiments.

Figure and ROI type	Channel 1 (RED)	Channel 2 (Green)	Manders Overlap Coefficient	M1 (% Red in overlap)	M2 (%Green in overlap)
1C (Whole worm)	SYX-17::mCherry	Mitotracker Green	0,8897 ± 0,009746	0,6442 ± 0,08361	0,5418 ± 0,07033
1C (Square)	SYX-17::mCherry	Mitotracker Green	0,9270 ± 0,006266	0,6448 ± 0,09119	0,6350 ± 0,08327
1D (Whole worm)	TOMM-20::RFP	VAMP-7::GFP	0,8657 ± 0,01616	0,1014 ± 0,03826	0,1543 ± 0,03785
1D (Square)	TOMM-20::RFP	VAMP-7::GFP	0,8844 ± 0,01490	0,1053 ± 0,02890	0,2876 ± 0,04862
1E (Whole worm)	TOMM-20::RFP	USO-1::GFP	0,9354 ± 0,01248	0,02520 ± 0,004283	0,05220 ± 0,01459
1E (Square)	TOMM-20::RFP	USO-1::GFP	0,9416 ± 0,01066	0,03540 ± 0,005115	0,1956 ± 0,04986
3A (Whole worm)	SYX-17::mCherry	GFP::LGG-1	0,8981 ± 0,01584	0,2441 ± 0,07076	0,1074 ± 0,03393
3A (Square)	SYX-17::mCherry	GFP::LGG-1	0,9160 ± 0,01347	0,3481 ± 0,07799	0,1283 ± 0,04317
3B (Whole worm)	dsRED::LGG-1	USO-1::GFP	0,9284 ± 0,01077	0,4630 ± 0,1193	0,2094 ± 0,06742
3B (Square)	dsRED::LGG-1	USO-1::GFP	0,9358 ± 0,007102	0,4468 ± 0,1296	0,2256 ± 0,05246
S2F (Square)	SYX-17::mCherry	USO-1::GFP	0,9040 ± 0,01532	0,1880 ± 0,04467	0,2985 ± 0,06687

Supplementary Table S4: Summary of oligonucleotides used in the present study.

Gene	Sequence	Enzymes for TOPO subcloning	Target vector
syx-17 RNAi Forward	CCCGGGATGTATGAAAAAACTGCGAATCG	SmaI-AgeI	pL4440
syx-17 RNAi Reverse	ACCGGTATCACTCGTGGCCGATCGTTTG	SmaI-AgeI	pL4440
uso-1 RNAi Forward	CTGCAGATGTTCGCTTATTCAACGTC	PstI-AgeI	pL4440
uso-1 RNAi Reverse	ACCGGTGGCTCGTCCTCATCATCTGTCA	PstI-AgeI	pL4440
nsf-1 RNAi Forward	GGATCCATGAGTCCAGTCCC	BamHI-KpnI	pL4440
nsf-1 RNAi Reverse	GGTACCGGACGGTACAAGTTTAGAG	BamHI-KpnI	pL4440
syx-17 promoter Forward	CTGCAGCGCCAATGACCCTTTTCTAA	PstI-XbaI	pPD96.75
syx-17 promoter Reverse	TCTAGATGAGCGTTTAGCAAGAAAATTTGGCC	PstI-XbaI	pPD96.75
syx-17coding region Forward	CCCGGGATGTATGAAAAAACTGCGAATCG	SmaI-AgeI	pPD96.75
syx-17coding region Reverse	ACCGGTATCACTCGTGGCCGATCGTTTG	SmaI-AgeI	pPD96.75
vamp-7 coding region Forward	GGATCCATGCCGGGCCGTGTGATGTCAGTG	BamHI-AgeI	pPD96.75
vamp-7 coding region Reverse	ACCGGTGGTATAACACCATTTTTGTGCAAAATTGTGAG	BamHI-AgeI	pPD96.75
uso-1 promoter Forward	CTGCAGTCGATTGAATAAAACTATCG	PstI-BamHI	pPD96.75
uso-1 promoter Reverse	TCTAGAATTTACCTGAAAACAATTATTG	PstI-BamHI	pPD96.75

uso-1 coding region Forward	CTGCAGATGTTCGCTTATTCAACGTC	PstI-AgeI	pPD96.75
uso-1 coding region Reverse	ACCGGTGGCTCGTCCTCATCATCTGTCA	PstI-AgeI	pPD96.75
snb-6 coding region Forward	GGATCCATGATTCATCAACAACTACCAGTGAG	BamHI-AgeI	pPD96.75
snb-6 coding region Reverse	ACCGGTGGGAGAAATGCGTATGCGATTCCGGCAA	BamHI-AgeI	pPD96.75
pmp-3 RT Forward	CTTGCTGGAGTCACTCATCGTGTTATG	-	For qPCR
pmp-3 RT Reverse	GTCGGGACGCTGATTTATCATCTTC	-	For qPCR
syx-17 RT Forward	CTATAATTGACCCTATCAGACAGC	-	For qPCR
syx-17 RT Reverse	GTTCCTTCATATCATCTGCCAAT	-	For qPCR
vamp-7 RT Forward	ACGATGTTCGGCAGGTGA	-	For qPCR
vamp-7 RT Reverse	GTGCAAAATTGTGAGCACAAT	-	For qPCR
uso-1 RT Forward	GAATTGAGCAGAGCCAACTCGCA	-	For qPCR
uso-1 RT Reverse	CGAGGCGAGCGACAAATTGATC	-	For qPCR
snb-6 RT Forward	CTACCAGTGAGTCGTATTGGTTGGGA	-	For qPCR
snb-6 RT Reverse	CAGCTCATCTCACGCTTGATCTTG	-	For qPCR
nsf-1 RT Forward	GGAAAGATGTTGAACGCGAGAGAG	-	For qPCR
nsf-1 RT Reverse	GGACTGATGAGCTTCCAGCCAT	-	For qPCR
